# Evaluating Modern Implicit Bias Training in the Urology Workforce

**DOI:** 10.7759/cureus.77677

**Published:** 2025-01-19

**Authors:** John M Myrga, Shyam Patnaik, Bishoy Gayed

**Affiliations:** 1 Department of Urology, University of Pittsburgh Medical Center, Pittsburgh, USA

**Keywords:** diversity training, education, equity, inclusion, urology

## Abstract

Introduction

Implicit bias can lead to unintended influence in the treatment of other people and impact the care of patients. As such, there has been an increase in training to improve provider awareness of their own biases. There are many ways to address bias training within the medical community, but we currently do not have data on its use within urology. Our current study examined the frequency, modality, and intensity of bias training among urology providers.

Methods

We created a survey to evaluate exposure to bias education in members of the urologic community, with an optional exercise in implicit bias training. This survey was sent to a nationwide audience through the Society of Academic Urology (SAU) database and also shared on social media. All responses were anonymous. Institutional review board approval was obtained prior to study commencement (IRB STUDY23020038). Patients were asked about their exposure to bias training within their institution, who provided that bias training, and if they felt bias training to be effective.

Results

Of the 84 providers who responded to the survey, 77 (91%) were physician providers (e.g., attendings), five (5%) were resident physicians, and two (2%) were advanced practice providers (APPs). Additionally, 56 (67%) respondents reported that bias training is mandatory within their departments, while 63 (75%) indicated that the primary method of training is an online course or module. Only seven (8%) respondents noted that their bias training was sponsored by the urology department or division at their institution. Only 37 (44%) providers agreed the bias training they were provided had a positive impact. While race and gender in the workplace are frequently presented, topics related to ageism, ableism, and diversity of religion are less often represented.

Discussion

Most of the providers surveyed had some exposure to bias training. The majority of training is presented as online courses or modules sponsored by the institution or university. These modalities appear to be associated with low satisfaction rates among urology providers. Bias training and recognition of biases are important for patient outcomes and satisfaction, especially within urology. To ensure we provide optimal care for our patients, ownership of this issue and ensuring adequate training for our providers is warranted.

Conclusion

Our study shows that bias training within the urologic community is limited in scope and its current implementation leads to low satisfaction among providers. This is the first study to evaluate this subject among urology providers. Future studies are warranted to evaluate more engaging and meaningful ways to educate providers on diversity topics.

## Introduction

Implicit bias in medicine is a topic of interest due to the unconscious effect bias plays in providing patient care. The National Institutes of Health defines implicit bias as unintentional attitude, behavior, or actions that impact decisions or behaviors of one social group of people over another [1]. Since individuals are unaware of these biases, they can lead to unintended influence in the treatment of patients on a level not recognized by the provider [2-4]. Studies have shown that there are disparities in medical outcomes of Black patients compared to Caucasian patients, possibly related to perceived racial bias [5]. While race is the most cited area of unconscious bias, gender, religion, age, and ableism are also areas highly impacted by provider biases. Healthcare providers are not impervious to these subconscious biases and their effects on patient care [6]. 

Increased focus has been placed on understanding how to implement bias recognition and training in the urology community. It is not currently known if urology providers are receiving training in bias recognition and its impact on patient care. Furthermore, we do not know which modalities are being utilized and if providers find them effective. Given the known issues of provider burnout and "click fatigue," the commonly used modality of module-based training may not be an effective method [7]. Understanding the exposure of the urologic community to bias training and their experience may prove important to its effective implementation.

One way to promote knowledge of these subconscious influences is through the implicit association test (IAT). This test is a computerized assessment that measures how long a participant takes to associate pictures or words representing a social group (e.g., Black or Caucasian people) with characteristics (e.g., good, stubborn) [8]. The use of the standardized IAT has led to the identification of implicit racial bias in the treatment of patients of color [9]. While specific training in implicit bias has improved awareness in training programs such as emergency medicine and OB/GYN, there is limited data on the evaluation of implicit bias in urology [10].

We aimed to assess exposure to formal bias training among urologists, with a secondary goal of evaluating exposure to the IAT within the urologic workforce. This will help address the knowledge gap in the field. The purpose of our study is to raise awareness of implicit racial bias in urology and improve standardized training on identifying and managing implicit bias.

## Materials and methods

We created a brief survey to evaluate exposure to bias training in members of the urologic community with an optional exercise in implicit bias. This survey has not previously been studied or validated. This survey was sent to a Canadian and United States audience, and all responses were anonymous. The University of Pittsburgh Institutional Review Board (IRB) deemed our study exempt from formal approval (IRB STUDY23020038).

Survey design

Our survey was created using Survey Monkey, allowing for anonymous responses that did not collect participants' personal or internet protocol (IP) information. Basic demographic information was obtained. The survey was intended to gauge participant exposure to bias training within their institution. Question formats included multiple choice, a drop-down menu, and free text fields. Patients were asked about their exposure to bias training within their institution, who provided that bias training, and if they felt the training to be effective. At the end of the survey, all participants were asked to complete the race IAT created by Project Implicit at Harvard University. Participation in this part of the study was optional. Participants were then asked to provide their final results from this exercise at the end of the study. The survey questions are included in the Appendices.

Survey distribution

We invited physicians, fellows, residents, advanced practice providers (APPs), nursing, and administrative staff within urology to complete the survey via various forms of social media with a sharable link. In addition, we sent the survey to all members subscribed to the Society for Academic Urology (SAU) email distribution service. This is made up of providers based in the United States and Canada. We also requested these members to recruit their fellow faculty, residents, and staff members to complete the study. There was no incentive for completion of the survey, and participation was voluntary. The survey was published and emailed via the SAU email server on June 19th, 2023, with a repeat email sent two weeks later. A link to the survey was also posted by the study creators on Twitter. We allowed for reposting and sharing of this link. The study was closed six weeks following the initial email response. Data was then abstracted from the Survey Monkey Collection site.

Data analysis

Data was downloaded from the Survey Monkey website and uploaded to an SPSS dataset (Aramonk, NY, Version 29, 2022). Descriptive data was then obtained and presented as appropriate. Some questions allowed for multiple responses, so data may not add to 100 percent. Graphs and figures were created using Microsoft Excel (2023 Edition). 

## Results

**Figure 1:**
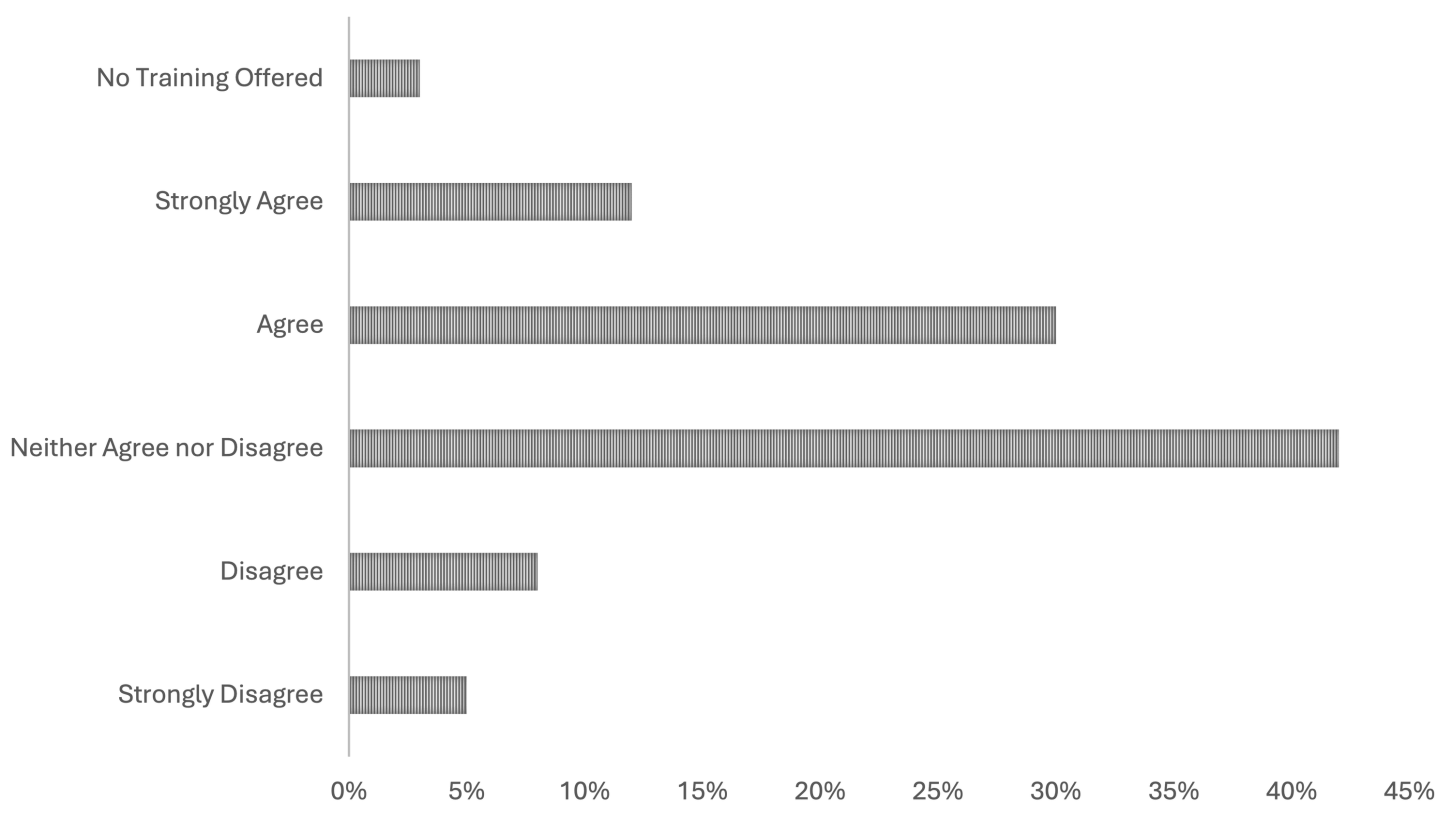
Percentage of providers who agree that their current diversity training had a positive impact on their education.

Of the 84 providers who responded to the survey, 77 (91%) were physician providers (e.g., attendings), five (5%) were resident physicians, and two (2%) were advanced practice providers (APPs). Additionally, 56 (67%) respondents reported that bias training is mandatory within their departments, while 63 (75%) indicated that the primary method of training is an online course or module. Only seven (8%) respondents noted that their bias training was sponsored by the urology department or division at their institution. Only 37 (44%) providers agreed the bias training they were provided had a positive impact (Figure 1).

**Figure 2:**
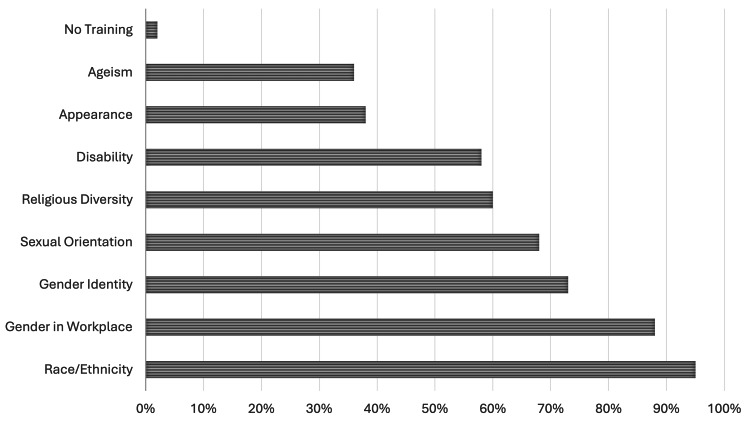
Provider-reported content of their current diversity training.

The most common topics reviewed in respondents’ implicit bias training were race and gender in the workplace (Figure 2). Compared to other topics, ageism, and physical appearance were less commonly discussed.

**Figure 3:**
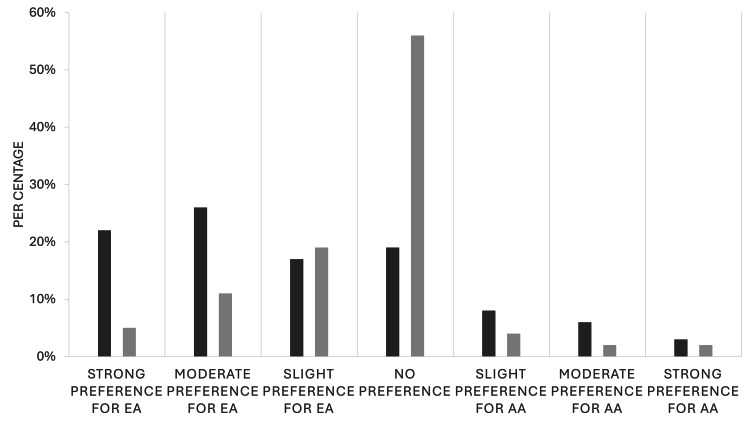
Results of the IAT in our urology population compared to the general population who completed the race IAT. Dark grey: results from a national comparison population; light grey: results from our urology-specific population EA: European ancestry; AA: African ancestry; IAT, implicit association test

We then examined the responses specific to the IAT. Forty-one (49%) providers had previously taken an IAT. Of the 84 providers, 55 (66%) chose to complete the racial IAT, and 42 (50%) participants elected to self-report the results of their IAT. Among those who completed the race IAT as part of the survey and chose to report their results, most showed little to no preference for European American over African American responses. The results of our study population are compared with reference values from a national, non-specific population that had taken the racial bias IAT (Figure 3).

## Discussion

This study aimed to evaluate the exposure of the urologic workforce to bias training within their institution. We had a secondary aim of evaluating the exposure of participants to the racial IAT. Our results were primarily limited to academic faculty and noted that over two-thirds had required bias training within their department. Over 75% noted that this training was provided through modules, and only 37 (44%) indicated that the training was useful. About half of providers were familiar with an IAT and 42 (50%) participants elected to take an IAT as part of our study.

Bias training and the importance of diversity, equity, and inclusion continue to be a topic of interest to the medical community and the urologic community as a whole [6]. What is not clear is how to best engage and inform providers of how bias can impact patient care. IATs have been used to help providers describe and understand their own biases [6]. It is important to know that while useful, these tests are not clear markers of bias and have their own limitations [11,12]. Modules have also been widely used to introduce and teach providers about bias and diversity, but their efficacy remains questionable [13,14]. Our study confirms that this is the most common modality used to inform and engage providers regarding diversity. We note that only 44% of people who responded noted this training had a positive impact on their training. While our study is not large enough to examine this specific question, module-based learning appears to have low satisfaction with diversity training in the urologic community.

We also noted that while there was a wide variety of topics that were covered, many important topics were left out of standard diversity training. Urology is a specialty that has wide exposure to patients of different races, ethnicities, religious backgrounds, and gender identities. This further highlights the need for urology to have this exposure and training to provide the best care for our patients. Our results show that there is currently a gap in the training offered to providers as well as a high dissatisfaction with their implementation. Further steps are needed to address this knowledge gap and improve provider satisfaction in their education.

One method that has been implemented across many surgical fields is the concept of cultural complications grand rounds. This has been pioneered by the University of Maryland and The University of Michigan. This is an in-person or virtual discussion that is led by a faculty member with a pre-designed curriculum around multiple topics related to diversity and cultural components of medicine. Early studies have shown that this approach may have increased satisfaction for providers and trainees [15,16]. This allows for a discussion-based approach allowing providers of all levels to discuss these issues in a clinical setting with their peers. While this is only one option, it may help to address the lack of exposure and high dissatisfaction seen in current studies. These interventions are simple and impactful methods to increase awareness of diversity within the urology community and avoid the click fatigue often associated with module-based methods. We believe our study brings awareness to this issue and allows for targeted direction for future studies.

Our study comes with some limitations. First, we polled the academic urology community so our results are limited to members within that community. "Furthermore, our population was limited to English-speaking North American providers. Understanding the exposure of providers in community-based settings or those based outside of North America would provide important information that is missing in our study. In addition, almost 90% of our responses were attending faculty. While this is important as this is where the majority of patient-facing care is completed, understanding the exposure of allied providers, trainees, and nursing staff would be greatly informative. Our study is also limited by the nature of the questions as well. Since our goal was to obtain general information, our survey is non-standardized. Our methods of outreach also were targeted at physicians using social media and those within the academic community. We aimed to keep our study brief to increase participation but further studies should probe deeper into specific aspects of diversity training in the urologic community.

## Conclusions

Our study shows that bias training within the urologic community is limited and its current implementation leads to low satisfaction among providers. Current methods, such as the IAT, are rarely used, and reliance on module-based learning may lead to low satisfaction. More engaging methods, such as the Cultural Complications Grand Rounds model, may be more effective and engaging for urology providers. Future studies should explore the use of these interventions and gain a broader understanding of bias training within the urologic community.

## Appendices

Implicit association test

We are researchers from the University of Pittsburgh Department of Urology aiming to better understand the exposure of the medical community to bias training/education. We are also examining responses from residents, fellows, and other medical providers regarding an implicit association test relevant to the medical community. These results are completely anonymous; however, we are asking for basic demographic information to better characterize exposure to diversity training. We encourage those interested to fill out the associated questionnaire and the two included tests and report their answers using this Google form. We hope that this will also provide information for participants to use in their own daily practice.

Urology-specific questions

1.        AUA section

2.        Age

3.        Gender identity

4.        How would you describe yourself?

5.        Training status

6.        Have you previously taken an implicit association test?

7.        Does your institution have required bias/diversity training or education?

8.        If so, how did you receive this information?

9.        What topics of information did you receive?

10.       Who sponsored this education/training?

Implicit association test

This portion of the survey requests that you complete one of the Harvard implicit association tests (IATs). We then ask that you self-report the results from the end of the IAT in this form. All test responses are anonymous, and data is only supplied to the Harvard IAT group and is not shared with our research study.

11.        Implicit association test: race

https://implicit.harvard.edu/implicit/takeatest.html - then proceed to IAT: race

Questions/concerns/comments

12.        Other comments/concerns
